# Open Letter to a Physician Who Has Inquired about Homœopathy

**Published:** 1894-05

**Authors:** Edward Cranch


### OPEN LETTER TO A PHYSICIAN WHO HAS INQUIRED ABOUT HOMŒOPATHY.

Dear Doctor:—The subject shall be explained to you, as you have inquired, and perhaps you will see why we are earnest in wishing all physicians everywhere to study and practice the truths of Homœopathy.

We hope to convince you of the reason, practicality, and honesty of our purpose, and to show you that a man can practice Homœopathy, and increase his means of cure, his ease of cure, and his certainty of cure, and at the same time be, in spite of innuendo and obloquy, in the enjoyment of that best of companions, a satisfied conscience. It really seems as if some were deterred by the fear that we were pretenders, were hornblowers, not believing what we profess, and only desirous of hoodwinking an easily deceived public. Fear of that same public prevents too violent abuse, which was tried on us at first, but which most distinctly failed; now we are simply "not recognized," an awful fate, 'tis true, but one that can be survived. You are brave enough to ask about this unrecognized system, you hope it may not be all a sham, and you want to know, you know.

You have perhaps read the article on "Homœopathy" and the notice under "Medicine," in the *Encyclopædia Britannica;* but ten to one you have not read the article "Homœopathy" in the Stoddard American Supplement to the same Encyclopædia written by an able lawyer, from a fair study of the best authorities. The former exhibit is full of errors and of a morbid desire to show up weak points and make capital of the same, the latter article is clear, concise, and without prejudice for or against, only a judicial statement of the case. We will not quote it here, nor refer to it further, but address ourselves to you colloquially, and try to satisfy your tentative inquiries.

You are a physician, you know what a physician's work is, and how it differs from that of a student, yet carries many student-habits along with it. You know that people come to you for information, for relief, for cure. They all want to know what ails them; some think they know, and are eager to tell it all; some want you to guess, some will not speak a word without a brace of questions for each letter of it; some are sweet and some are silly; some are too suspicious and secretive; some are too confidential and loquacious; but they all, without exception, want to know what ails them, what is this, what does that mean, what makes this do so, and so on, often before you have time to grasp a tenth part of the real situation—hence you must be, and doubtless are, well up in anatomy, physiology, pathology, and diagnosis, you can look at the *arcus senilis* and shake your head over the vista of interior fatty degeneration that used to be thought inseparable from that phenomenon.

When you can put your finger on the pulse and from its wayward action, and compressible weakness, argue that the heart needs a tonic, you can outline the areas of dullness, whose boundaries you cannot change, you can tell by the râles, blowing, and murmurs, all that is physically changed in the heart and lungs; you put your thermometer under the tongue and decide the presence or absence of fever. From the anamnesis you elicit the constitutional diatheses that befog your patients' systems, the scrofulous, syphilitic, catarrhal, nervous, asthmatic, dropsical, asthenic, and alienist cases are duly sorted out, each with its chapter of heredity and its horoscope of prognosis.

You find out all this and more, the urine yields its secrets, the bacteria tell their stories, the aspirator draws off serous fluids with their characteristic cells and chemical reactions, every art of the expert examiner and cross-examiner of every part, brings every divergence from health to view, and establishes a more or less thorough diagnosis. Sometimes it is possible to name the condition, sometimes it must be described by circumlocution or periphrasis, but there must always be a ready name or brief title, for the patient, so that his or her friends may be duly informed, and the doctor's wisdom or his mistakes published to all concerned and a few more. How large a part of this matter of diagnosis plays is known from the customary remarks of patients: "So-and-so can cure me, I know, for he knows *just what ails me;*" or this other, "He didn't seem to know what was the matter, so I was afraid to take his medicine." Now this, you say, has nothing to do with Homœopathy! Hold on, just here is where it comes in.

Is your duty *done* when you have found out much or little about the patient, and correctly named his disorder, and told him what to expect if it goes on its way unchecked? Of course not; and you are very properly indignant at the question. No, the next thing is the prescription, for which you put on your wisest air, and, if you have read Cathell, you pause just before you put down the fifth or sixth ingredient, though it be only a *corrigens* or a simple article that you have already chosen, and you lift your pen in air, while you ask, with the closest solicitation, some new question, and, nodding cheerfully or sadly when you get the answer, as if you had expected it just so, you proceed to note down the next line as if it were just determined on. Very telling effects can be worked up in this way, only you have to submit your prescription to the eye of the patient, or nurse, who may happen to guess, or know, what you put down so gravely, and if it is Syr. Aurant., Cort. q. s., or Aqua fort., ℥ iv, may have Latin enough to read it and get on your track.

Banter aside, we all study effect, thereby striving to arouse confidence and hope, most potent auxiliaries of all practice.

But the homœopath can do it so much more neatly, for he can select his dose from his neat, odorless pocket-case, drop a portion from a clean paper on the patient's tongue, rouse no disgust, run up no bills for the patient at the drug-store, be certain of the purity and strength of his own drugs, and so far happy.

You may carry your own doses, but you must disguise them in sugar-coats, cordial draughts, pills not always easy to swallow, powders often bitter and repulsive to the last degree. Do you begin to see where Homœopathy comes in?

So far we may still jest, but now the more serious aspect of the case comes on.

In your diagnosis of the disease, let us suppose a case of erysipelas, easily recognized and treatment laid down in fair characters, that he who runs may read. "Antipyretic and supporting;" very good, but the means, the drugs. In your early days you heard of the muriated Tincture of Iron, supported by Quinine, and aided further by Calomel, "to defibrinate the blood," but iron ruled the roost for erysipelas. Iodine or Nitrate of Silver, was used externally to "limit the disease." *Now*, according to W. Gilman Thompson in Pepper's text-book, we learn that many cases get well alone, that all local applications but the most gentle are injurious, that antipyretics do harm by inducing depression, that the strictest cleanliness must be observed to limit contagion. Stimulants and iron are strongly recommended. All very good in their way, but is there not more to be desired; in abbrevating the course of the disease, in preventing complications and sequelæ, and so insuring a more rapid convalescence?

Your treatment overlooks many striking points made use of by us in distinguishing between cases apparently similar. The very points that are the most important for diagnosis, those to which we turn before we say this is not erythema, or scarlatina, or measles, or rötheln, but erysipelas, these points are the *least* valuable in deciding on the remedy, that is to say, the remedy for this particular case, that differs from that one yonder, not so but what both are erysipelas, but so as to call for a different drug. You recognize the difficulty, of course, and you meet it with more or less of the stimulant, or you withhold this or that drug that you think will disagree, but why? Have you any law beyond the general law of empiricism? How many caustics, plasters, poultices, and unguents, how many antipyretics, alteratives, tonics, purges, baths, and lotions have been tried on erysipelas, and are still used with no law but experience to guide! Observe the phrase, "no law but experience," do not we hear you say, what is the crank talking about? Is not that enough?

It sounds well, but it is *not* enough. It is true in a way, because all laws are proved by experience, but in applying them do we always appeal to experience? Is experience alone a safe guide without some formulating law? And when law is discovered and applied, then experience can be guided, nay, foretold with assurance. Did experience guide Columbus to a new world? All past experience had seemed to demonstrate that the earth was flat, and fixed under the sun and stars. His discoveries demonstrated the laws of its shape and motion, and made *new discoveries* possible.

That is what Homœopathy claims, that it is a new discovery, a demonstration of existing law in the domain of medicine—the law that *all actions and reactions are equal and opposite.* The application of this law leads us to give Belladonna to cases of erysipelas whose eruption resembles most nearly the eruption of that drug; Rhus-tox., Croton-tiglium, Bee poison (Apis), Sulphur in cases whose appearance and general state, as manifested in all observable symptoms, agree with the known appearances and states induced upon the healthy by those drugs.

In general, Belladonna antidotes (because it can cause) smooth erysipelas, with the highest fever; Rhus-tox., the finely vesicular variety; Croton, the coarsely vesicular; Apis, the œdematous; Sulphur, the maculated, etc. The direct action of these drugs is directly resisted by the organism, and the original health returns, by vital reaction, when the drug is expelled. Vital reaction is *always* trying to throw off disease; it does it more promptly if stimulated in its very centres by a minute dose of a drug that acts directly in the line of its effort. It is like putting an obstacle in the way of an angry man, by which you stimulate him to further effort, and he will free himself if the obstruction be not too great. So the dose must not be strong enough to paralyze action, or the angry man, the disease, finding himself at bay, rages more violently and does more damage till stunned or stupefied by the surgical operation of a club or the temporary suppression of an anodyne.

In either case the house is not cleared of the intruder, who lies low for a fresh disturbance.

Homœopathy claims to shorten curable disorders, to reach many diseases heretofore incurable, and to simplify all prescribing by the observation of a natural law. Is it not worth investigation? Do not hold off, and refuse to look through the Galilean telescope, but come forward, read, study, prove by experiment. Shorten your cases, ease them without narcotics, cure them before you find out what ails them, feel the satisfaction that comes from greater certainty of results, and do not let the extra study needed frighten you.

Remember that Hahnemann, without ever having seen a case of cholera, said that Camphor, Arsenic, Copper, and Veratrum-album, not in combination, but singly and in small dose, as occasion called for them, would be most powerful and sufficient remedies, and that they have so proven—that one man, practicing in the public hospitals and barracks of Italy cured three hundred and seventy-seven consecutive cases of true Asiatic cholera, so pronounced by many competent observers. The small dose and its non-repetition while improvement continues, be it one hour, or one day, or one month, or one year, are necessary corollaries of the unvarying law that reaction always follows action. Just as the acknowledgment of a fault is a necessary step to its abandonment, so a touch on the disease, in the very path of the disease, is needed to rouse vital reaction when tardy or at fault.

If all constitutions were perfect, no drugs would ever be needed, but if a stimulus to recovery must be applied, don't break down the wall to get at it, but find the way the disease entered, and send in your little messenger along the same route, and both will come back together.

In conclusion, let me recommend you to read the works of the master; do not be deterred by his scolding, or by vagueness of theory in places, search out the true merit, it is there, and will amply repay you, as thousands and thousands can testify.

In a future letter we will tell you of some of the details of study and application of the law.

EDWARD CRANCH, M. D.

